# Postmortem interval estimation of human skeletonized remains through luminol chemiluminescence

**DOI:** 10.1007/s00414-024-03343-8

**Published:** 2024-10-18

**Authors:** Catarina Ermida, Joana Rosa, Eugénia Cunha, Maria Teresa Ferreira

**Affiliations:** 1 Department of Life Sciences, Centre for Functional Ecology (CFE), Laboratory of Forensic Anthropology, University of Coimbra, Calçada Martim de Freitas, Coimbra, 3000-456 Portugal; 2 Department of Chemistry, Molecular Physical-Chemistry R&D Unit, University of Coimbra, Coimbra, 3004-535 Portugal; 3 Department of Life Sciences, Research Centre for Anthropology and Health (CIAS), University of Coimbra, Calçada Martim de Freitas, Coimbra, 3000-456 Portugal; 4 National Institute of Legal Medicine and Forensic Sciences (INMLCF), South Branch, Rua Manuel Bento de Sousa 3, Lisboa, 1150-334 Portugal

**Keywords:** Human skeletal remains, Postmortem interval, Luminol, Chemiluminescence, Forensic Anthropology

## Abstract

Postmortem interval (PMI) estimation represents a significant challenge in the forensic sciences, particularly when dealing with human skeletal remains. A screening protocol for distinguishing possible remains of forensic interest is a crucial tool for judicial purposes. In this context, luminol chemiluminescence emerges as a promising method, with low overall costs and required time. This method is primarily used as a presumptive test, based on the understanding that the intensity of the chemiluminescence reactions decreases with an increase in the postmortem interval, thus underlining its practical implications.

This research aims to expand previous research on the potential of luminol chemiluminescence, evaluating its usefulness in estimating PMI. Our sample comprised 239 human clavicles, with known PMI. The luminol solution was sprayed on each powder bone sample in a dark room, observed by the naked eye and photographed. The intensity of the chemiluminescence reaction was measured using a binary and a 5-level scale.

The present results reveal that this method is a suitable tool for PMI estimation as a presumptive test, reducing time and costs in criminal investigations. The findings underscore the high sensitivity of luminol chemiluminescence for detecting recent PMI but also highlight a notable incidence of false positives. Thus, our results confirm luminol chemiluminescence as a powerful tool for dating time of death, particularly for identifying forensic relevant remains. Still, the relatively low specificity indicates that it should be complemented with additional tests for further confirmation and scientific validation of the remains’ forensic relevance.

## Introduction

Estimating the postmortem interval (PMI), which corresponds to the amount of time elapsed since death until the body is found, as correctly as possible, can impose a significant scientific challenge, especially when dealing with skeletonized remains [1]. A primary consideration, when skeletonized human remains are recovered in suspicious circumstances, is to distinguish whether or not the material has forensic significance, in which case it would merit further judicial investigation by law enforcement agencies [2–4]. One crucial issue to be discussed, and which provides additional difficulties to this task, is the definition of forensic interest in terms of time, as the differentiation between forensic and archaeological relevance can have a different legal time frame, according to specific national laws (15/20 years, 50 or even 100 years). This fact highlights how challenging establishing time since death can be, as it demonstrates the difficulty of developing reliable methods that can predict postmortem intervals of forensic interest. Hence, even though distinguishing between recent findings with forensic or historical significance poses numerous challenges, forensic anthropologists are called upon to articulate their assessments following certain criteria, such as the Daubert criteria, thereby needing to support their statements using scientifically validated methods, which compel the forensic anthropologists to employ the most precise and analytical approaches [5, 6]. These approaches ensure that the assessment of the PMI of the human remains is unaffected by environmental variations, and the outcomes consistently align with scientific data [6]. However, validating methods is highly complex since the majority of the PMI estimation techniques can be affected by numerous environmental factors, meaning that ‘‘Variability in the decay is the rule’’ [7–10]. Due to bone structure and rigidity, its decomposition rate is slower than soft tissue decomposition. Still, it can be affected by many influencing factors, such as weathering, scavenging, and diagenesis. Diagenesis, which corresponds to the ion exchange between bone and its surrounding location, can be influenced by intrinsic bone characteristics such as size and structure and factors like temperature, pH, soil, humidity, and the organic content of the soil [11–13]. However, this contamination mostly concentrates on the bone surface [14]. Also, various factors such as cost, time limitations, the origin of the remains and their preservation state, and the decomposition conditions add complexity to forensic investigations [6]. For this reason, there is a need for the development of an affordable, easy, and fast technique capable of accurately differentiating between forensic interest and archaeological bones [13].

Estimating the time since death typically begins with a thorough examination of skeletal remains at a macroscopic level, with a subsequent microscopic analysis of a bone Sects [6, 9]. The time of death can also be dated by assessing the concurrent degradation of additional elements found at the discovery site (such as clothing, personal items, etc.) or by employing chemical and physical techniques [15]. Several investigations are documented in the literature regarding the estimation of the postmortem interval. Although, despite numerous studies attempting to develop reliable PMI estimation methods, dating skeletonized human remains continues to be a complex task for forensic anthropologists [1, 8, 15]. Currently, only isotope dating methods, like radiocarbon, appear to offer reliable information for estimating the time since death of skeletonized human remains. However, this analysis comes with expensive costs and elaborate methodologies [16].

The use of luminol chemiluminescence for estimating the time elapsed since death was proposed by Introna et al. (1999). This technique has garnered considerable attention as a presumptive test for distinguishing between forensic relevant and archaeological bones since it constitutes a simple and affordable method [17]. Although presumptive tests are commonly considered initial indicators, demanding further confirmation with more robust techniques, they are essential in forensic practice.

**Fig. 1:**
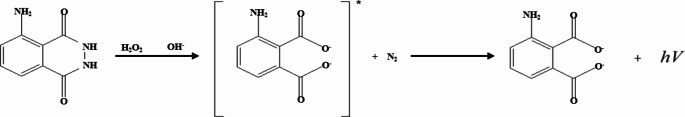
The general mechanism for the chemiluminescence reaction of luminol (adapted from Ermida et al., 2023 [17])

Luminol (5-amino-2,3-dihydro-1,4-phthalazinedione), a cyclic acyl-hydrazide, is considered one of the most essential chemiluminescence systems (which refers to the emission of light from a chemical reaction), and it was discovered by Albrecht in 1928 [8, 18–20]. The luminol reaction results in the formation of molecular nitrogen and an excited 3-aminophthalate dianion, being the chemiluminescent attributed to the radiative deactivation of the 3-aminophthalate dianion from its singlet excited state (Fig. 1.), resulting in the subsequent emission of blue light [20, 21]. This complex process can be influenced by multiple factors, such as temperature, pH, and ionic strength [19].

The luminol reaction has been widely used for over 80 years in forensic contexts to detect blood traces after Specht (1937) investigated the role of hemin in this reaction and its potential application [22]. This technique is based on spraying a standard mixture of luminol, solved in an aqueous solution containing hydrogen peroxide, on the blood traces, making them visible through the emission of a blue light resulting from the chemiluminescence oxidation of luminol when the traces come into contact with the solution, on account of its high sensitivity to hemoglobin [13, 17, 21, 23–25]. The process exploits luminol’s ability to react with a catalyst (the iron ions present in hemoglobin), resulting in chemiluminescence emission when exposed to an oxidizing agent such as hydrogen peroxide. Thus, when luminol is applied, ferric heme groups in hemoglobin catalyze both the decomposition reaction of the hydrogen peroxide and the luminol oxidation [13, 19, 21, 25, 26].

Previous investigations demonstrate its enormous sensitivity for detecting blood traces seemingly invisible to the naked eye, even with a dilution range from 1:100000 to 1:5000000 [8, 14, 26, 27]. Another advantage of this technique is its repeatability, as the luminol solution can be used multiple times over bloodstains, allowing chemiluminescence repetition [19]. However, it is essential to note that false positive reactions are possible to occur due to potential interference from substances that can trigger chemiluminescence emission, such as animal hemoglobin, sodium hypochlorite (household and industrial bleach), disinfectants or antiseptics with potassium permanganate or iodine, iron, copper, some furniture polishes and enamel paints, some motor vehicles’ interior fabric or plant peroxidases (turnips, horseradishes, parsnips) [23, 24, 26, 28]. Nevertheless, experienced forensic practitioners claim that it is possible to distinguish interfering substances from haemoglobin in some specific cases by evaluating some parameters visible to the naked eye (duration and intensity of the emission or spatial distribution), like when those are solid, producing a different pattern of distribution, or resourcing to spectroscopic equipment, through a quantitative evaluation of emission spectra, when there is a significant spectral shift of the chemiluminescence catalysed by some substance comparing to the haemoglobin [19, 23].

More recently, several studies extended the use of this method for estimating time since death in skeletal remains, correlating PMI with the presence of hemoglobin traces in bone tissue [8, 9, 15, 25]. After death, the blood supply nourishing the skeleton ceases. Still, the rigid nature of bone, due to its elevated mineral content, can safeguard the remaining hemoglobin in a relatively stable state, as long as the structural integrity of the bone is maintained. Over time, the amount of hemoglobin in the skeletonized remains will decrease, reducing the intensity of the luminol reaction when the test is applied. Consequently, assessing the hemoglobin content within skeletonized remains emerges as a viable method for estimating the time since death, as the chemiluminescence reaction intensity decreases with an increasing postmortem interval [8, 9, 13, 14, 27].

This technique has its value attested in the literature, as it is a fast, easy to perform and to interpret (even by the naked eye with no sophisticated equipment needed), and inexpensive method [6, 8, 9, 27, 28].

In 1999, Introna and colleagues obtained promising results regarding estimating human skeletal remains through luminol chemiluminescence. In this study, the authors recorded the reaction in a dark room using a TV camera and measured the presence or absence of chemiluminescence using a greyscale, revealing a potential objective quantitative correlation between time since death and chemiluminescence in powdered bone, as the reaction’s intensity increased with the decreasing PMI. According to this study, a positive result was consistently obtained in recent bones, whilst a negative reaction was commonly observed in older bones. In Ramsthaler’s and co-authors’ research (2009; 2011), it was concluded that negative results (lack of chemiluminescence) seamed more reliable and of greater significance. They proposed that more expensive and elaborate radionucleotide methods could be restricted to luminol-positive results, indicating a forensic-relevant PMI. Creamer and Buck (2009) tried luminol method across different bones from the same individual and found no variability among the elements. On the other hand, Caudullo and team (2017), which also studied the influence of using different types of bones on luminol testing, concluded that this method should be carefully used on non-intact bones and bones other than long bones. Sarabia et al. (2018), presented a direct, low-cost complementary technique, using a luminometer to accurately measure luminol chemiluminescence in relative light units (RLU), achieving precise quantification of data to distinguish between remains of forensic interest to those that are not. The authors concluded that this new approach could be very helpful for estimating time since death, as a good degree of precision can be obtained.

Although many studies have been developed regarding this method, there is still a need for a more comprehensive sample range, with smaller PMI intervals, to establish an accurate technique, which can be applied in forensic practice following Daubert criteria.

This work aims to expand previous research by evaluating the potential of luminol chemiluminescence as a cost-effective and reproducible technique for estimating the postmortem interval. In that regard, a wider sample, with more detailed intervals, was tested with luminol, providing statistically significant information concerning this technique, and bringing the use of this method closer to forensic practice, according to Daubert criteria. Additionally, this study investigates the effectiveness of this technique in distinguishing skeletal remains that hold forensic relevance from those that do not.

## Materials and methods

**Fig. 2:**
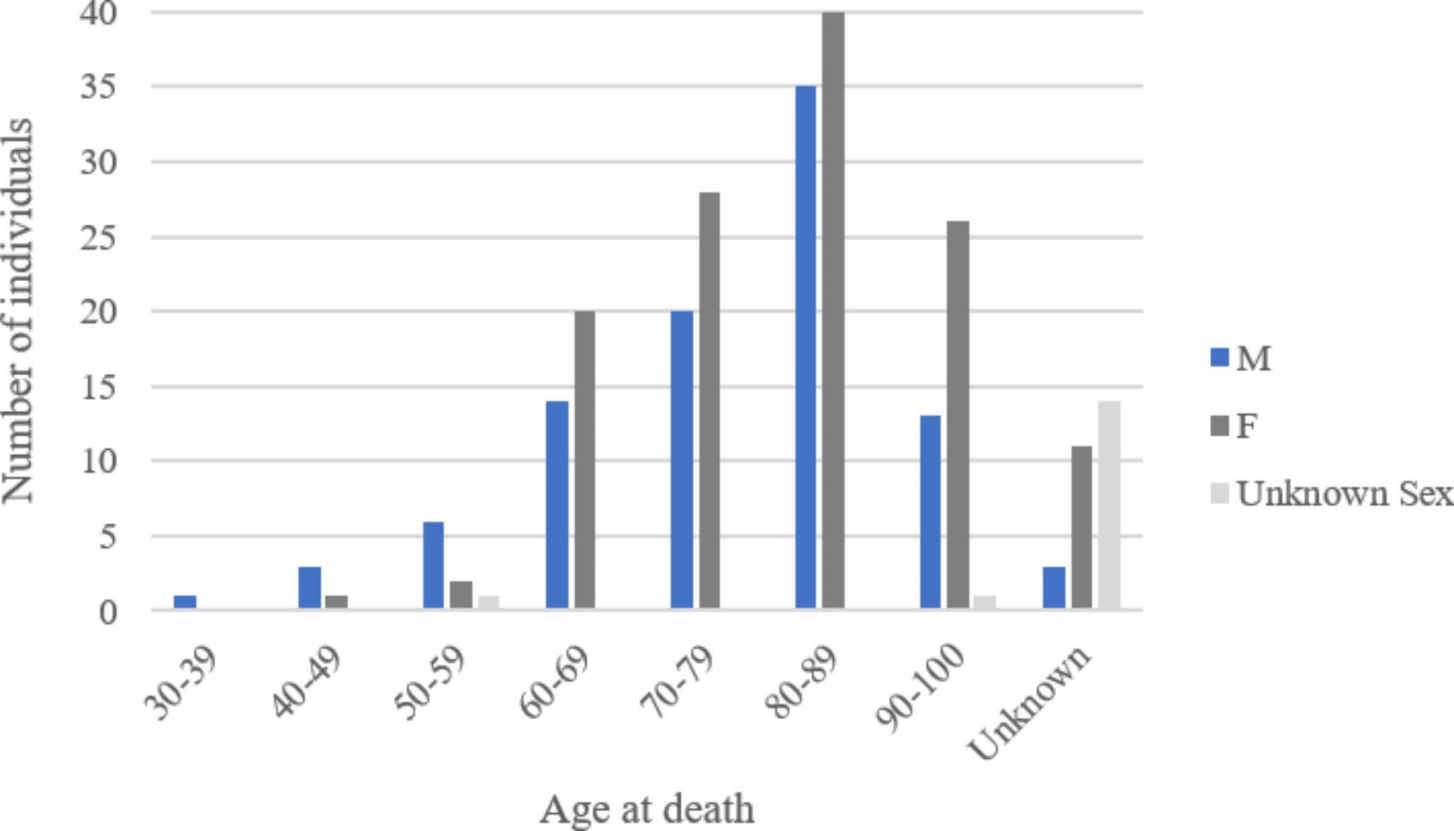
Sample distribution according to sex and age

Our work was composed of a sample of 239 human clavicles, from adult individuals of both sexes (see Fig. 2 for details), collected from different origins: fresh bones from autopsies performed at the Portuguese National Institute of Legal Medicine and Forensic Sciences (INMLCF.IP.), Centre Branch (approval from the Ethics Commission of the Portuguese National Institute of Legal Medicine and Forensic Sciences - CE-010/2018) (*n* = 7); dry skeletal remains from the 21st Century Identified Skeletal Collection [29] (*n* = 197); dry skeletal remains from autopsies performed in the 20th century at the INMLCF.IP., South Branch (approval from the Ethics Commission of the Portuguese National Institute of Legal Medicine and Forensic Sciences - CE-21/2021) (*n* = 26) and dry skeletal remains from the Valle da Gafaria’s osteoarcheological collection [30] (*n* = 9). The sample comprised individuals with ages at death ranging from 37 to 100 years (mean: 78.78, median: 81), distributed as evenly as possible between both sexes (male - mean: 77.23, median: 81, min: 37, max: 99; female - mean: 80.13, median: 81, min: 43, max: 100) and across PMI intervals. The sample’s known PMI ranged between 2684 days and 560 years, corresponding to the time interval between death and the luminol application. Table 1 illustrates the sample distribution according to PMI-defined intervals. The decision to focus exclusively on clavicles, without ante or perimortem trauma or identified pathology, was to ensure the sample’s standardization.

**Table 1:** Sample division according to PMI

PMI (years)	Number of individuals
5 - <10	13
10 - <15	111
15 - <20	43
20 - <25	49
25 - <30	2
30 - <40	4
40 - <50	6
50 – 60	2
> 100	9

The subsample obtained from fresh cadavers during the autopsies performed at the INMLCF.IP. Centre Branch (after checking RENNDA – National Register of Non-Donors – and receiving approval from the Ethics Commission of the Portuguese National Institute of Legal Medicine and Forensic Sciences – CE-010/2018) was composed of fresh clavicles which were previously prepared (reduced to powder) and used by our team in a previous study [27]. Until the necessary conditions for the study were met, the clavicles were preserved by freezing at 4 °C in properly identified zip-lock bags, and then defrosted, water macerated and manually cleaned resourcing to sandpaper and brushes. For the reduction into powder of this subsample, a small portion of each clavicle was extracted with a power saw, pulverized in a liquid nitrogen mill and collected in an Eppendorf tube (50 mg). This subsample (*n* = 7), included in the 5–10 years PMI interval, was the only one in which the bones were not buried.

The remaining sample was also cleaned and prepared with brushes and sandpaper. The current study attempted to reduce diagenetic influence, focusing the analysis on cortical bone, through careful and meticulous sample preparation. A scalpel was used to reduce into powder, and 50 mg of powder for each clavicle was collected in a labelled Eppendorf tube.

After preparing the solution of Luminol 16oz according to the reagent’s directions, the technique was applied in an entirely dark room, spraying the luminol (at room temperature) onto each sample, placed on a clean white paper sheet. A drop of blood was used as a positive control. It was demonstrated that luminol exhibits thermal instability [19], so precautions were taken in order to protect the solution from high temperatures. The solution was applied at room temperature and used for no longer than 3 h after preparation. The sample treatment and the luminol application were conducted entirely by the same operator. To mitigate the potential irritant effects of luminol, and considering its application in aerosol, the operator used appropriate protective gear such as gloves and protective clothing during the spraying process.

**Fig. 3:**
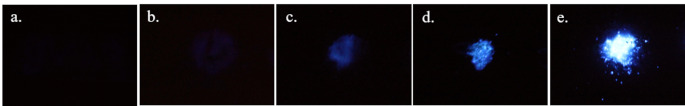
5-level scale interpretation for the luminol chemiluminescence reaction (**a**. negative; **b**. barely positive; **c**. weak positive; **d**. positive; **e.** strong positive)

The observation occurred in situ, by the naked eye in total absence of light, simultaneously with the chemiluminescence reaction, and the results were immediately recorded. Each chemiluminescence reaction was also photographed with a Canon EOS 500D (F/5, 30 s, ISO 3200, 27 mm) (Canon Inc., Tokyo, Japan) at the moment of the reaction. A binary scale (positive or negative) and a 5-level scale (referring to the increasing degree of chemiluminescence) were used to measure the intensity of the reaction (see Table 2; Fig. 3 for details). During the observations, the samples were anonymized, ensuring that the observer remained unaware of the postmortem interval.

**Table 2:** 5-level scale interpretation for the luminol chemiluminescence reaction. (adapted from Ermida et al., 2017 [27]).

Result	Interpretation
Negative (-)	No visible chemiluminescence to the naked eye.
Barely Positive (+)	The reaction is barely visible to the naked eye.
Weak Positive (++)	The reaction is visible with low intensity to the naked eye.
Positive (+++)	The reaction is easily visible to the naked eye.
Strong positive (++++)	The reaction is strongly visible to the naked eye.

Statistical analysis was performed using RStudio software, and Microsoft Excel, to assess the value of luminol chemiluminescence technique as a time since death estimation diagnostic tool, through the evaluation of sensitivity (True Positive Rate - TPR), specificity (True Negative Rate – TNR), and other parameters such as FPR (False Positive Rate), FNR (False Negative Rate), PPV (Positive Predictive Value), NPV (Negative Predictive Value) and DOR (Diagnostic Odds Ratio), considering different cutoff values [31]. Cutoff values of less than 100 years, less than 50 years, and less than 30 years were considered. A 100 years PMI corresponds, by convention, to the time limit between non-historical and historical human remains, and 30–50 years PMI constitutes the maximum legal threshold for human remains to be considered of forensic relevance in the majority of the countries. The inclusion in the present study of a cutoff value of 80 years was initially planned, as the results obtained by Introna et al. (1999) pointed out that bones with PMI higher than 80 years showed no chemiluminescence. However, it was not possible to include clavicles with those PMI values in our sample.

The statistical analysis did not consider variations related to sex and age-at-death, aligning with previous literature [8, 9, 13]. However, a comparison was made between the overall sample results and the results differentiated by sex. The assessment of the inter and intraobserver error was not performed in the present research since it was already evaluated in a prior study [17], which included a larger sample.

## Results

The results obtained from the luminol technique are summarized in Tables 3 and 4. As an important note, the blood sample used as positive control showed the strongest chemiluminescence reaction observed in the entire experiment.

The analysis of the luminol test using a binary scale demonstrated positive results (+) in all the samples up to the 25–30 years interval (Table 3). For PMI greater than 30 years, positive results were more irregular, presenting 75% of positive results in the 30–40 years interval and once more 100% positive results in the 40–50 year interval. For the PMI interval exceeding 100 years, 66.67% of the samples still tested positive. On the other hand, negative results (-) were only observed in samples with PMI larger than 30 years.

**Table 3:** Luminol chemiluminescence results by PMI intervals (in years) for the binary scale

PMI:	5 - <10	10 - <15	15 - <20	20 - <25	25 - <30	30 - <40	40 - <50	50 – 60	> 100
-	0	0	0	0	0	1 (25%)	0	1 (50%)	3 (33.33%)
+	13 (100%)	111 (100%)	43 (100%)	49 (100%)	2 (100%)	3 (75%)	6 (100%)	1 (50%)	6 (66.67%)

The 5-level scale (Table 4) provided a more detailed breakdown of luminol chemiluminescence across increasing PMI. The results showed that “strong positive” results (++++) were only obtained in the samples up to 20 years PMI. On the contrary, “barely positive” results were only shown on postmortem intervals greater than 30 years. The 5–10 years interval samples showed stronger chemiluminescence reactions, presenting exclusively strong (++++) and positive (+++) results. Samples with times since death larger than 25 years revealed uniquely “weak positive” or weaker chemiluminescence results (“barely positive” and “negative”). For the 30–40 years interval, results varied with 25% “negative”, 50% “barely positive”, and 25% “weak positive”, suggesting a declining trend in chemiluminescence intensity, corroborated by the results obtained in the following larger intervals.

**Table 4:** Luminol chemiluminescence results by PMI intervals (in years) for the 5-level scale

PMI:	5 - <10	10 - <15	15 - <20	20 - <25	25 - <30	30 - <40	40 - <50	50 – 60	> 100
-	0	0	0	0	0	1 (25%)	0	1 (50%)	3 (33.33%)
+	0	0	0	0	0	2 (50%)	4 (66.67%)	1 (50%)	6 (66.67%)
++	0	38 (34.23%)	18 (41.86%)	28 (57.14%)	2 (100%)	1 (25%)	2 (33.33%)	0	0
+++	9 (69.23%)	51 (45.95%)	22 (51.16%)	21 (42.86%)	0	0	0	0	0
++++	4 (30.77%)	22 (19.82%)	3 (6.98%)	0	0	0	0	0	0

**Table 5:** Luminol chemiluminescence results by sex and PMI intervals (in years) for the binary scale

PMI:	5 - <10		10 - <15		15 - <20		20 - <25		25 - <30		30 - <40		40 - <50		50 – 60		> 100	
	F	M	F	M	F	M	F	M	F	M	F	M	F	M	F	M	F	M
-	0	0	0	0	0	0	0	0	0	0	0	0	0	0	1	0	2	0
+	5	8	64	41	19	18	29	20	0	1	1	2	2	3	0	1	5	1

**Table 6:** Luminol chemiluminescence results by sex and PMI intervals (in years) for the 5-level scale

PMI:	5 - <10		10 - <15		15 - <20		20 - <25		25 - <30		30 - <40		40 - <50		50 – 60		> 100	
	F	M	F	M	F	M	F	M	F	M	F	M	F	M	F	M	F	M
-	0	0	0	0	0	0	0	0	0	0	0	0	0	0	1	0	2	0
+	0	0	0	0	0	0	0	0	0	0	1	1	1	2	0	1	5	1
++	0	0	21	14	7	10	17	11	0	1	0	1	1	1	0	0	0	0
+++	3	6	33	16	10	8	12	9	0	0	0	0	0	0	0	0	0	0
++++	2	2	10	11	2	0	0	0	0	0	0	0	0	0	0	0	0	0

According to Tables 5 and 6, no significant differences were detected between the results obtained from female and male samples. Both sexes showed similar results, consistent with previous studies, suggesting that sex may not affect the outcome of the luminol test.

Regarding the different cutoff values, 2 of the 239 clavicles with a time since death of less than 100 years and 1 with a PMI of less than 50 years tested negative for the luminol reaction. Concerning the Cutoff < 30 years, no false negative results were obtained for the luminol chemiluminescence method. On the other hand, unexpected false positive results occurred for all the different cutoff values (6 of the 9 bone samples for the cutoff < 100 years; 7 of the 11 bone samples for the cutoff < 50 years; 16 of the 21 bone samples for the cutoff < 30 years). Overall, a correct classification of 96,7% for the cutoff values of < 100 years and < 50 years, and 93,3% for the cutoff < 30 years was demonstrated. Table 7 presents diagnostic statistics for the luminol chemiluminescence method as a postmortem interval estimation tool using different cutoff values. The sensitivity of this method in our sample, for the different cutoff values, achieved almost perfect results (cutoff < 100 years: 0.991; cutoff < 50 years: 0.996; cutoff < 30 years: 1). However, the specificity was estimated to be much lower (cutoff < 100 years: 0.333; cutoff < 50 years: 0.363; cutoff < 30 years: 0.238), derived from the high percentage of false positive results. The Diagnostic Odds Ratio obtained was 57 for the cutoff of less than 100 years, and 129.7 for the 50-year cutoff, indicating enhanced diagnostic accuracy. This parameter could not be calculated for the less than 30 years PMI cutoff, since there were no false negative results.

When considering a cutoff value of less than 100 years, categorizing the sample as non-historical, the positive predictive value, corresponding to the probability of having a PMI inferior to 100 years when the chemiluminescence reaction is positive, is 0.974. Yet, the probability of rejecting a relevant case is 0.40. In opposition, the ratio of truly negative samples from all those with negative results (NPV) is 0.60, and the proportion of samples incorrectly classified as positive from all the positive obtained results (FDR) is 0.026. Reducing the cutoff value to a PMI of less than 50 years results in an increase in the NPV to 0.8, and, consequently, a decrease in the relative risk of false rejection to 0.20 Regarding a time since death of less than 30 years, closer to the forensic threshold of many countries, there were no false negative results, as previously mentioned, meaning that all the chemiluminescence reactions with a negative result were truly negative samples. However, the FDR is slightly higher for this cutoff value, as more false positive results were obtained. These results demonstrate the potential efficacy, and limitations, of luminol chemiluminescence as a diagnostic tool for PMI estimation assessment, with performance varying across different PMI intervals and cutoff values.

**Table 7:** Diagnostic statistics for the assessment of the luminol technique as a PMI estimation method given different cutoff values. (TPR - true positive rate; FPR - false positive rate; TNR - true negative rate; FNR – false negative rate; PPV - positive predicted value; FRR – false rejection rate; NPV - negative predicted value; FDR – false discovery rate; DOR - diagnostic odds ratio)

Cutoff Value	*P*	TPR	FPR	TNR	FNR	PPV	FRR	NPV	FDR	DOR
< 100	0.96	0.991	0.667	0.333	0.009	0.974	0.4	0.6	0.026	57
< 50	0.95	0.996	0.636	0.363	0.004	0.970	0.2	0.8	0.030	129.714
< 30	0.91	1	0.762	0.238	0	0.932	0	1	0.068	- ^a^

^a)^ This value cannot be calculated since there are no false negative results for the cutoff value of 30 years

## Discussion

Establishing an accurate PMI estimation has important legal implications, as well as identifying the forensic relevance of a case. However, dating time of death of human skeletal remains constitutes a major challenge in forensic sciences, even when the aim is merely to differentiate those with forensic interest from archaeological remains, due to the countless number of factors that can affect the decomposition process. The developed methods do not offer precise estimations or require expensive, complex, and time-consuming applications. Therefore, numerous techniques have been recently studied in order to determine an easier and more suitable method for completing this task. This research aims to thoroughly explore the potential of the luminol chemiluminescence technique for estimating time since death in a robust sample with a large range of PMI intervals, using different forensically relevant cutoff values.

The analysis of the luminol chemiluminescence results across different postmortem intervals reveals this method as a suitable tool, as a presumptive test, for time since death estimation. Although some unexpected results exposed positive reactions in samples with dates of death older than 100 years, suggesting that the luminol technique can detect hemoglobin in historic skeletal human remains, significant differences were found in the chemiluminescence intensity through the different time intervals, pointing out the decreasing intensity of the chemiluminescence reactions with time. According to the 5-level scale analysis, intense chemiluminescence reactions, described as “strong positive” results, were solely observed in the first three intervals (5 to 20 years PMI). It is also worth noticing that, even in these lower PMI intervals, the most common reaction is the “positive” reaction [69.23% (5–10 years); 49.95% (10–15 years); and 51.16% (15–20 years) versus 30.77%; 19.82%, and 6.98% for the “strong positive”, respectively]. The decrease in “positive” reactions as the PMI intervals increase is clearly noticeable, being replaced by a majority of “weak positive” reactions, achieving 100% of the results in the 25–30 years interval. From the 30–40 years interval to more than 100 years PMI, a very faint light-reaction (“barely positive”) appeared in 13 of 21 clavicles, whereas in 3 (30–50 year PMI) a “weak positive” was observed, and in the remaining 5 no reaction was detected.

These results are mainly consistent with the existing literature. Although the great number of false positives in PMI over 100 years was not expected (> 50% of false positives), it was previously described in other studies. Ramsthaler et al. (2009) described an occurrence of false positive results in 7.5% of the cases. In previous research (2011), Ramsthaler and team reported a percentage of false positive results of 30%. Later, Capella et al. (2018) recorded an even higher number of ancient bones testing positive for luminol (41.1%), closer to our obtained results. Still, in most of these studies, including the present one, the small sample size for PMIs over 100 years should be considered cautiously. The primary issue associated with a high percentage of false positives, usually related to low forensic relevance cutoff values, is the pursuit of cases either irrelevant or with no forensic interest, wasting valuable time in examinations and further techniques. On the contrary, Introna and colleagues’ (1999) experiment disclosed an almost complete absence of chemiluminescence in samples older than 50 years (1 out of 10 samples showed a slightly faint reaction), concluding that no chemiluminescence reaction occurs on bone samples with PMI of 80 or more years. The observed differences in the results within studies may be explained by numerous reasons, such as the burial context storage conditions of the samples, or even the variations in the experimental design.

The proportion of false negative results constitutes a variance between our study and the previous investigations mentioned above. Out of the 230 samples, 2 were false negatives, considering the cutoff < 100. This value drops to 1 false negative result for the cutoff < 50 and 0 for the cutoff < 30. Even though false positive and false negative results should be given equal importance, from a scientific perspective, from a forensic context point of view, false negatives carry the potential danger of erroneously ruling out a forensically relevant case, closing the investigation.

The diagnostic performance of luminol chemiluminescence was evaluated using different cutoff values, reflecting different forensic relevance thresholds, as the diagnostic utility of a technique should always consider how forensic relevance is defined. The high TPR and PPV across all cutoffs (< 100, < 50, <30 years) indicate the test’s strong ability to correctly identify positive results. However, the low TNR values suggest a lower specificity due to the significant number of false positives. These results express that, although the luminol technique is effective at identifying recent cases, its reliability decreases for older remains due to lower specificity. DOR value for the cutoff < 100 showed reasonable diagnostic accuracy, being improved for the cutoff < 50. This value was not calculated to the cutoff < 30 due to the absence of false negatives, indicating robust performance. These findings suggest that luminol chemiluminescence is a valuable tool for estimating postmortem interval, particularly differentiating between recent and archaeological remains. Its high sensitivity makes it suitable for initial screenings, but the relatively low specificity advises the need for complementary tests to confirm results.

The usage of a 5-level scale for the PMI estimation through the luminol technique revealed substantial differences between PMI interval groups. According to our results, “barely positive” results were consistently exclusively recorded for PMI values over 30 years. On the other hand, “strong positive” and “positive” reactions were only observed in samples with no more than 20 or 25 years since death, respectively. These results highlight the potential of this technique when an intense or nearly faint chemiluminescence reaction is observed, as these results are never coincident in the same PMI range. However, a “weak positive” result was observed in a wide range of times since death (from 10 to 50 years), therefore highlighting the need for other techniques to corroborate the result and narrow the time range.

In effect, despite its wide use in forensic practice, the luminol chemiluminescence test usually requires further examination. It should be employed alongside other methods, like the evaluation of the inorganic components in bone tissue. If the results from these simple and faster methods align, it is likely that the estimation can be considered reasonably accurate. When there are no agreements in the estimated time interval, a posterior confirmation using more expensive methods that evaluate radioisotope levels in bone tissue (mainly radiocarbon dating) is needed. Combining methods represents a promising solution, especially for cases involving multiple fragments. In these cases, radiocarbon testing is unfeasible for economic reasons, so a preventive screening protocol should be applied in order to identify the elements worth testing. In forensic practice, due to the complexity of the decomposition process, a combination of methods is advisable, and the selection of these must rely on each case’s aspects.

The main disadvantage of the luminol technique, as with the vast majority of the methods intended for estimating the postmortem interval, relates to the number of factors that can influence the results obtained by this method. The primary limitations arise from the environmental burial conditions and diagenetic processes that can influence the decomposition rate, accelerating the degradation of the bone matrix, causing false negative results, or, on the opposite, slowing the decomposition process or mimicking positive reactions (interfering substances) even when no haemoglobin is present, revealing false positive results [9, 13, 15, 23]. Also, Caudullo et al. (2017) reported that the reliability of this technique in dating the time of death may be conditioned by the types of bone used, and its integrity (intact or more damaged). A previous work conducted by our team (Ermida et al. 2023a), aimed at accessing the influence of temperature, humidity, pH, and some soil types on the PMI estimation through the luminol test, concluded that these taphonomic factors can influence the results obtained through this technique. Therefore, this evaluation must be managed carefully, always considering the context in which a body is found. In the present study, the different origins of the collected samples did not allow the decomposition context to be considered in the analysis of the results, as it was unknown. Additionally, although the type of bone used was standardized, the level of bone preservation was not always the same, with some clavicles in the sample group being more damaged than others during the powder reduction process. Future research should focus on further investigating the impact of various factors on luminol chemiluminescence, such as environmental and taphonomic conditions to which the body has been exposed, its state of preservation, as well as intrinsic cadaver factors (e.g., cause of death or antemortem pathologies), which may affect haemoglobin degradation and consequently the luminol reaction [25]. Preparation procedures, the circumstances of the recovery, and storage conditions may also affect the outcome [8, 27]. Therefore, it is essential to normalize the entire procedure. Ideally, all samples should be stored uniformly until the method is applied, and the protocol should be thoroughly standardized.

The fact that a small portion of the sample (*n* = 7) came from autopsy cases may be considered a limitation of our research. Ideally, the entire sample would originate from the same location and include bones buried under the same conditions. However, we believe that this methodological difference did not affect the results, as they were similar to those obtained from buried bones with the same PMI.

Another limitation inherent to our study is the unequal distribution of the sample across the different postmortem interval ranges, along with the lack of individuals with PMI closer to the date of death. However, for ethical and judicial reasons, gathering human remains for scientific experiments can constitute a challenge. So, it was unattainable to increase the number of individuals for this sample. Hence, our team gathered all the possible clavicles with older/smaller postmortem intervals from the available collections, for which we obtained approval from all required entities, including ethics committees. In future research including a sample better distributed through the different PMI intervals would be valuable to yield even more robust results. Regardless of the outlined limitations, our work presents a robust sample, both in size and composition, with 239 individuals distributed for a wide range of PMI intervals.

Despite the mentioned limitations, luminol chemiluminescence presents many advantages supporting its widespread usage in forensic casework. Our research confirms it as a simple, fast, easy-to-interpret, and economical method. Technical aids, like digital image capture, are not mandatory since previous studies concerning the assessment of this technique intra and interobserver error by visual means, attest to its validity as a reliable and reproducible procedure, practically independent of observation bias, supporting its potential as a rapid diagnostic test [8, 17]. The new data presented in the current work adds important findings to the applicability of this technique. As many countries’ forensic thresholds to classify a case as of forensic relevance are lower than 30 years, the absence of false negative results for this cut-off value corroborates the pertinence of luminol chemiluminescence employment for distinguishing whether the remains are of forensic interest or not, further emphasizing the importance of the results arising from this investigation. Additionally, the noncoincidence between the “strong positive” and “positive” results up to 25 years PMI and the “barely positive” results from 30 years PMI onward represents an advancement in the understanding of this technique, as well as increased reliability in the application of the 5-level scale.

These important results bring this method closer to its acceptance and practical application in the legal setting. Still, some work remains for the luminol chemiluminescence technique to fully comply with the mandatory Daubert Standards [32], which ensure the quality, reliability, and replicability of the methods to be included in a scientific testimony in court. Despite being widely accepted for blood detection and meeting the criteria for testability and peer review, this technique application for PMI estimation is less established, needing further validation. However, studies like the present one provide valuable data, contributing to its recognition. According to our results, this methodology is reliable for PMI values up to 30 years, with clear trends in chemiluminescence intensity supporting its forensic application. It demonstrates high sensitivity but lower specificity, indicating the need for standardized protocols to reduce false positives. Thus, when evaluated against Daubert Standards, although this method meets several criteria, it still requires further refinement to enhance its diagnostic reliability.

This study corroborates what was previously reported in the literature, suggesting that the results achieved by this method must be interpreted with caution. Thus, according to our research, we cannot support the conclusion that luminol chemiluminescence is suitable for estimating time since death as a single method. Our findings demonstrate that a negative luminol test result, confirming a PMI of no forensic relevance, is more conclusive than a positive luminol test result. However, the almost absence of false negatives increases the interest in this technique as a presumptive test.

## Conclusion

The present manuscript provides an insightful analysis of the effectiveness of the luminol chemiluminescence technique for postmortem interval estimation. The obtained results suggest this method is a useful tool for assessing time since death, and support its use in forensic investigations, particularly for distinguishing between recent and archaeological remains. However, despite its high sensitivity, its specificity decreases for longer postmortem intervals. Thus, caution is advised when applying this technique due to the potential false positive results.

As for future perspectives, besides those already mentioned throughout the manuscript, the main goal should be the development of a standardized, reliable protocol for estimating the time since death through luminol chemiluminescence. In this regard, chemiluminescence reaction intensity quantification procedures like the ones proposed in previous studies [14, 15], resorting to instrumental techniques, should be further investigated since it allows for precision. Nevertheless, the postmortem interval estimation must always rely on a combination of complementary methods. Based on our current knowledge, the luminol chemiluminescence technique alone cannot accurately estimate the postmortem interval. Therefore, investigating the combination of this test with other diagnostic methods can be essential to establish a reliable screening protocol for estimating this crucial parameter in forensic science.

## Data Availability

All data generated or analysed during this study are included in this published article.
